# The Demographic Profile of Colorectal Cancer Patients in Indonesia: Insights from a Single Center Experience and Exploration of Immune Response and Survival Outcomes

**DOI:** 10.12688/f1000research.155021.1

**Published:** 2024-11-14

**Authors:** Cosphiadi Irawan, Findy Prasetyawaty

**Affiliations:** 1 Division of Hematology and Medical Oncology, Department of Internal Medicine, Faculty of Medicine Universitas Indonesia-Cipto Mangunkusumo National General Hospital, Jakarta, 10430, Indonesia

**Keywords:** colorectal cancer, demographics, tumor characteristics, immune score, survival

## Abstract

**Background:**

Understanding the demographics, tumor characteristics, genetic mutations, and immune scores in colorectal cancer (CRC) patients may aid in tailoring treatment and predicting survival.

**Methods:**

This retrospective cohort study assessed clinical parameters, immune scores, and their relationship with survival in patients with CRC.

**Results:**

The study included 74 patients, mean age 53.7 years, mostly male (53.3%) and aged 41-70 (77.3%). Common comorbidities included cardiovascular diseases (29.3%) and hypertension (21.3%). Adenocarcinoma (74%) primarily affects the colon (73%). KRAS mutations and Microsatellite instability-High (MSI-H)/deficient mismatch repair (dMMR) were found in 1.3% and 16% of patients, respectively. Stage IV (77.3%) and liver metastases (52.7%) were prevalent. Immune score was influenced by cancer stage (p = 0.04) and metastasis (p=0.05). The immune score was not associated with survival (p = 0.181). Patients with comorbidities had lower one- (p = 0.027) and two-year survival rates (p = 0.037) survival rates. Cardiovascular comorbidities negatively impacted one-year survival (p = 0.047) and two-year survival (p = 0.037). The mean survival time was shorter for males (2.047±0.288 vs. 2.781±0.195 years, p = 0.041), patients with comorbidities (1.772±0.371 vs. 2.702±0.188 years, p = 0.017), and cardiovascular comorbidities (1.558±0.316 vs. 2.685±0.207 years, p = 0.038). Comorbidities (unadjusted hazard ratio [HR] 2.948, p = 0.023) and cardiovascular comorbidities (unadjusted HR 2.695, p = 0.046) were initially associated with survival but lost significance after adjusting for confounding variables.

**Conclusions:**

This study provides insights into CRC patient demographics and their interplay with the immune score and survival.

## 1. Introduction

According to GLOBOCAN 2020, colorectal cancer (CRC) stands as the third most predominant cancer globally, adding 10% of new cases in 2020, totalling over 1.9 million instances. Despite its ranking, CRC ranks second in terms of mortality rate, claiming 935,000 lives in 2020. It primarily affects the colon and the rectum. Geographic disparities in incidence and mortality rates are notable, with Europe and Australia/New Zealand reporting the highest incidence rates, whereas Eastern Europe experienced the highest mortality rates. Incidence rates are approximately four times higher in fully developed countries than in transitioning nations, although mortality rates exhibit less variability owing to increased fatalities in transitioning countries. ^1^

CRC often remains asymptomatic until its advanced stages, but its frequent symptoms include diarrhea, constipation, bloody stools, abdominal discomfort, unexplained weight loss, fatigue, and iron deficiency. Generally, affecting people over the age of 50 years, risk factors comprise a family history of CRC, Lynch syndrome, familial adenomatous polyposis (FAP), previous CRC diagnosis, or certain types of polyps. Lifestyle choices, such as eating habits rich in processed meats, low fruit and vegetable intake, smoking, obesity, sedentary behavior, and excessive alcohol consumption, further elevate the risk. This underscores the importance of adopting a healthy lifestyle and undergoing regular screenings, particularly for individuals over 50 years old or with a family history, to mitigate CRC risk. ^1^

Understanding the demographics of patients with CRC is crucial for identifying high-risk individuals and customizing screening and prevention strategies. Furthermore, delving into histopathology, tumor staging, location, and metastasis offers valuable insights for treatment planning and prognosis in CRC cases. Additionally, investigating genetic mutations in KRAS, NRAS, and BRAF is essential for guiding targeted therapy administration, predicting treatment responses, and determining prognosis. ^2^ Assessing the prevalence of Microsatellite Instability-High (MSI-H)/Deficient Mismatch Repair (dMMR) or Microsatellite Stable (MSS)/Proficient Mismatch Repair (pMMR) is crucial for predicting responses to immunotherapy and prognosis. ^3^ Understanding the percentage of patients receiving surgical treatment, targeted therapy, chemotherapy, and immunotherapy is essential for identifying areas in which therapeutic approaches can be improved.

The American Joint Committee on Cancer/Union Internationale Contre le Cancer (AJCC/UICC) TNM staging system is the primary method for classifying CRC, but lacks immune-based parameters, despite the known impact of the immune system on patient survival. However, the most up-to-date edition of Digestive System Tumors by the WHO emphasizes the importance of immune response in CRC diagnosis, highlighting the prognostic power of the immune score assay. ^4^ This standardized scoring system, determined by the concentration of CD3+ and CD8+ T cells in both the tumor core and the surrounding invasive margin, offers a more clinically significant prognostic value than TNM staging alone. ^5^ It helps estimate recurrence risk, guide treatment decisions, and assess responses to immunotherapies. ^6^ Immune score may aid in selecting patients for adjuvant chemotherapy, identifying candidates for neoadjuvant therapy, and predicting outcomes more accurately. ^7^ Notably, it categorizes patients into prognostic groups, indicating their risk of relapse and offering the potential for tailored treatment strategies in patients with CRC. ^8^

The objective of this study was to investigate the demographic and clinical characteristics of CRC patients, including age, sex, body mass index (BMI), comorbidities, tumor location, tumor side, KRAS, NRAS, BRAF mutation status, microsatellite instability, immune score, disease stage, presence of metastasis, and targeted therapy use. Additionally, this study aimed to investigate the relationship between patient characteristics and immune scores, as well as patient characteristics and survival.

## 2. Methods

This study was carried out at a tertiary care cancer facility, utilizing data from the Siloam Hospital database spanning 2020-2022. The dataset encompassed demographics, histopathology, tumor characteristics, staging, mutation status, treatment, comorbidities, and metastasis details. The study included adult patients (aged 18 years or older) with a confirmed diagnosis of CRC, with available clinical data from 2020 to 2022, and information on immune score and/or survival outcomes. Patients with other cancers or incomplete treatment records from outside the study period or different institutions were excluded. All CRC patients diagnosed and treated at the hospital between 2020 and 2022 were considered for inclusion. Given the limited number of CRC patients (n=75) during this period, all eligible patients who met the inclusion criteria were selected. The predefined follow-up period for the study was 3 years from the date of diagnosis or initiation of treatment. Survival outcomes were tracked through medical records, with regular follow-up visits documented in the hospital database. CRC diagnosis was confirmed via biopsy and histopathology with staging based on the American Joint Committee on Cancer (AJCC) Staging System 8 ^th^ edition. KRAS, NRAS, and BRAF mutations were identified using polymerase chain reaction (PCR). To assess the Immunoscore, two consecutive slides from formalin-fixed, paraffin-embedded tumor samples were stained with anti-CD3 and anti-CD8 antibodies and counterstained with hematoxylin. The slides were digitized and specialized software was used to measure the abundance of CD3+ and CD8+ T cells in the tumor core (CT) and invasive margin (IM). The software identified the tissue, delineated the CT area, and displayed the IM. The classification of CT and IM was validated by a pathologist, and the software excluded necrotic or artifact areas. A histogram validates the staining quality and serves as an internal quality control. The intensity of CD3CT, CD3IM, CD8CT, and CD8IM cells were translated into percentiles, and the mean of these percentiles formed the immunoscore. ^6^ The hospital's medical record team managed the database to ensure accurate and updated information. The study size was determined based on an expected 3-year overall survival (OS) rate of 75%, with a 10% margin of error and a 95% confidence level. Using a standard sample size calculation for proportions, the minimum required sample size was estimated to be 72 patients. Descriptive analysis, Kolmogorov-Smirnov tests for data normality, chi-square tests, Fisher’s Exact Test, and Kaplan-Meier Analysis were performed using SPSS software to explore the demographic and clinical characteristics of CRC patients. Cox regression was used to identify unadjusted and adjusted hazard ratios. This study was approved by the Ethics Committee of the Faculty of Medicine, Universitas Indonesia.

## 3. Results

### 3.1 Demographics of CRC patients

A total of 74 patients with CRC were treated at Siloam Hospital between 2020-2022 ( Table 1). The mean age at diagnosis was 53.7 years with the majority (77.3%) being 41-70 years old, and a slight majority being male (53.3%). Body Mass Index (BMI) analysis revealed that 38.7% of patients had a normal weight (18.5–22.9 kg/m ^2^) according to the WHO classification for Asia-Pacific. Notably, 33.3% were obese (≥25 kg/m ^2^), 16% were underweight (<18.5 kg/m ^2^), and 10.7% were overweight (23–24.9 kg/m ^2^). Comorbidities were present in 36% of the patients, with cardiovascular diseases being the most prevalent (29.3%), and hypertension present in 21.3% of patients. Other cardiovascular diseases included diabetes mellitus type 2, coronary arterial disease, dyslipidemia, hypertensive heart disease, and arrhythmia. Additionally, a few patients had other comorbidities such as benign prostate hyperplasia, hepatitis B, and asthma.

**Table 1.** Characteristics of sample population.

Age	
Mean ±sd	53.7 ±12.7
≤29 (%)	3 (4.1)
30-39 (%)	7 (9.5)
40-49 (%)	15 (20.3)
50-59 (%)	25 (33.8)
60-69 (%)	17 (23)
70-79 (%)	6 (8.1)
80-89 (%)	1 (1.4)
Sex,n (%)	
Male	49 (52.7)
Female	35 (47.3)
Body Mass Index based on Asia-Pacific classifications, n (%)	
Underweight (<18.5 kg/m ^2^)	12 (16.2)
Normal weight (18.5–22.9 kg/m ^2^)	29 (39.2)
Overweight (23–24.9 kg/m ^2^)	9 (12.2)
Obese (≥25 kg/m ^2^)	24 (32.4)
Comorbidities, n (%)	
Present	26 (35.1)
Cardiovascular Disease	21(28.4)
Hypertension	15 (20.2)
Dyslipidemia	2 (2.7)
Coronary arterial disease	4 (5.4)
Diabetes Mellitus type 2	6 (8.1)
Hypertensive Heart Disease	1 (1.4)
Arrhythmia	1 (1.4)
Benign Prostate Hyperplasia	1 (1.4)
Asthma	2 (2.7)
Hepatitis B	1 (1.4)
Absent	48 (64.9)
Location, n (%)	
Colon	54 (73)
Rectum	20 (27.4)
Side, n (%)	
Left	64 (86.5)
Right	8 (10.8)
No data	2 (2.7)
KRAS, n (%)	
Mutant	1 (1.4)
Wild-type	50 (67.6)
No data	23 (31.1)
NRAS, n (%)	
Mutant	-
Wild-type	49 (66.2)
No data	25 (33.8)
BRAF, n (%)	
Mutant	-
Wild-type	43 (58.1)
No data	31 (41.9)
MSI, n (%)	
Microsatellite instability-High (MSI-H)/deficient mismatch repair (dMMR)	12 (16.2)
Microsatellite stable (MSS) /proficient mismatch repair (pMMR)	28 (37.8)
No data	34 (45.9)
Immune score, n (%)	
High	24 (32.4)
Intermediate	7 (9.5)
Low	43 (58.1)
Stage, n (%)	
III	11 (14.9)
IV	59 (78.4)
No data	5 (6.8)
Metastasis, n (%)	
Present	58 (78.4)
Absent	12 (16.2)
No data	4 (5.4)
Targeted therapy, n(%)	
Yes	32 (42.7)
No	37 (50)
No data	5 (6.8)

Regarding the site of CRC, the colon was the most commonly affected area, affecting 54 patients (73%), followed by the rectum (27.4%). Locations of CRC in the colon area were observed in the sigmoid colon (24.0%), rectosigmoid (21.3%), and descending colon (10.7%), ascending colon (6.7%), cecum (2.7%), hepatic flexure (1.3%), anal region (1.3%), transverse region (1.3%), and anorectal region (1.3%). Histopathological examination revealed that adenocarcinoma as the predominant subtype (74%), followed by mucinous adenocarcinoma (9.5%). Most tumors were located on the left side(86.7%). Of the left-sided tumors, 53.1% were male. The proportion of female and male patients with right-sided tumors was equal. Among left-sided CRC patients, 88.5% of them had exhibited adenocarcinoma and 11.5% had mucinous adenocarcinoma. Among right-sided CRC patients, 85.7% presented with adenocarcinoma, and only 14.3% had mucinous adenocarcinoma.

KRAS mutations were detected in 1.3% of patients and absent in 68% of patients. NRAS mutations were absent in 66.7% of patients, and BRAF mutations were absent in 57.3% of cases. Overall, the prevalence was 16%, while MSS/pMMR prevalence was 37.3%. Notably, 33.3% of patients had a high immune score, 9.3% had an intermediate score, and 4% had a low score. Most patients were diagnosed with stage IV (77.3%), followed by 14.6% with stage III. The study found that 77.3% of patients had metastasis, primarily observed in the liver (52.7%), lungs (21.6%), and peritoneum (10,8%). Metastasis was also observed in the bone, ovarium, perineum, mesentery, pelvis, peritoneum, soft tissue perianal, bladder, omentum, uterus, vagina, bone, and para-aortic lymph nodes.

Most participants underwent laparotomy resection of the tumor. Chemotherapy was administered to most of the patients. The most commonly used regimens were FOLFOXIRI, XELOX, and FOLFOX. Targeted therapy was administered to 42.7% of patients, while 50.7% did not receive targeted therapy. The targeted therapies included Bevacizumab, Cetuximab, Regorafenib, and Ramucirumab. Immunotherapy was not administered to any of the patients in this study.

Forty-three patients (58.1%) had KRAS wildtype and left-sided tumors. Among them, 25.6% received cetuximab as targeted therapy, while 51.2% of them did not receive targeted therapy. MSS/pMMR was present in 37.8% of patients, and all of them received chemotherapy.

### 3.2 Relationship between patient characteristics and immune score

There were significant relationships between cancer stage and immune score (p = 0.04), as well as between metastasis and immune score (p = 0.05). Age, sex, body mass index, comorbidities, cardiovascular comorbidities, tumor location, tumor side, microsatellite instability (MSI), and KRAS mutations were not significantly associated with immune score. A detailed breakdown of the relationship between clinical parameters and immune scores is provided in Table 2.

**Table 2.** Patient characteristics and immune score relationship.

Patient characteristics	Immune score			p-Value *
	High	Intermediate	Low	
Age, n (%)				0.768 ^b^
<60 (%)	20 (40.0)	7 (14.0)	23 (46.0)	
≥60 (%)	7 (29.2)	0 (0.0)	17 (70.8)	
Sex, n (%)				0.188 ^a^
Male	10 (25.6)	6 (15.4)	23 (59.0)	
Female	14 (41.2)	1 (2.9)	19 (55.9)	
Body Mass Index based on Asia-Pacific classifications, n (%)				0.652 ^a^
Underweight	2 (16.7)	1 (8.3)	9 (75.0)	
Normal weight	11 (37.9)	3 (10.3)	15 (51.7)	
Overweight	3 (33.3)	1 (11.1)	5 (55.6)	
Obese	8 (33.3)	2 (8.3)	14 (58.3)	
Comorbidities, n (%)				0.206 ^b^
Present	6 (23.1)	2 (7.7)	18 (69.2)	
Absent	18 (37.5)	5 (10.4)	25 (52.1)	
Cardiovascular Comorbidities, n (%)				0.287 ^a^
Present	4 (19.0)	1 (4.8)	16 (76.2)	
Absent	18 (37.5)	5 (10.4)	25 (52.1)	
Location, n (%)				0.774 ^b^
Colon	17 (31.5)	5 (9.3)	32 (59.3)	
Rectum	7 (35.0)	2 (10.0)	11 (55.0)	
Side, n (%)				0.422 ^a^
Left	22 (34.4)	6 (9.4)	36 (56.3)	
Right	1 (12.5)	1 (12.5)	6 (75.0)	
MSI, n (%)				0.166 ^a^
Microsatellite instability-High (MSI-H)/deficient mismatch repair (dMMR)	5 (41.7)	4 (33.3)	3 (25.0)	
Microsatellite stable (MSS) /proficient mismatch repair (pMMR)	19 (67.9)	3 (10.7)	6 (21.4)	
No data	0 (0.0)	0 (0.0)	34 (100.0)	
KRAS, n (%)				1.000 ^a^
Mutant	0 (0.0)	1 (100.0)	0 (0.0)	
Wild-type	24 (48.0)	6 (12.0)	20 (40.0)	
No data	0 (0.0)	0 (0.0)	23 (100.0)	
NRAS, n (%)				-
Wild-type	24 (49.0)	7 (14.3)	18 (36.7)	
No data	0 (0.0)	0 (0.0)	25 (0.0)	
BRAF, n (%)				-
Wild-type	24 (55.8)	7 (16.3)	12 (27.9)	
No data	0 (0.0)	0 (0.0)	31 (100.0)	
Stage, n (%)				**0.040** ^a^
III	6 (60.0)	0 (0.0)	4 (40.0)	
IIIb	1 (100.0)	0 (0.0)	0 (0.0)	
IV	16 (28.1)	6 (10.5)	35 (61.4)	
No data	1 (16.7)	1 (16.7)	4 (66.7)	
Metastasis, n (%)				**0.050** ^a^
Present	16 (27.6)	6 (10.3)	36 (62.1)	
Absent	7 (58.3)	1 (8.3)	4 (33.3)	

* To determine the p-value, the immune score was classified into two categories: Low+Intermediate and High.

^a^ Fisher’s Exact Test

^b^ Chi-Square Test For Independence.

### 3.3 Relationship between patient characteristics and survival

The relationship between patient characteristics and survival indicated that overall comorbidities and cardiovascular comorbidities significantly affected the survival rates ( Table 3). For patients with comorbidities, the one-year survival rate was 56.3% compared with 87.5% in those without comorbidities (p = 0.027), and the two-year survival rate was 18.8% compared with 50.0% in those without comorbidities (p = 0.037). The mean survival time of patients exhibiting overall comorbidities was 1.772 ± 0.371 years, which was significantly lower than 2.702 ± 0.188 years) (p = 0.017). Similarly, patients with cardiovascular comorbidities had a one-year survival rate of 53.8% in comparison to 85.7% in those without cardiovascular comorbidities (p = 0.047), and a two-year survival rate of 15.4% compared to 48.6% in those without cardiovascular comorbidities (p = 0.037). The mean survival time of patients with cardiovascular comorbidities was 1.558 ± 0.316 years, which was significantly lower than 2.685 ± 0.207 years) (p = 0.038). Furthermore, survival was significantly linked to sex, with mean survival times of 2.047 ± 0.288 years for males and 2.781 ± 0.195 years for females (p = 0.041). There was no significant correlation between survival and variables, such as age, BMI, tumor location, tumor side, MSI, stage, metastasis, or immune score.

**Table 3.** Patient characteristics and survival relationship.

Patient characteristics	1-year survival		2-year survival		Survival time	
	Survived N (%)	p-value ^a^	Survived N (%)	p-value ^a^	(Mean ± SE, years)	p-value ^b^
Age						
<60 years	25 (78.1)	1.000	13 (40.6)	0.835	2.599 ± 0.241	0.213
≥60 years	12 (75.0)		6 (37.5)		1.963 ± 0.229	
Sex, n (%)						
Male	17 (68.0)	0.119	9 (36.0)	0.597	2.047 ± 0.288	0.041
Female	20 (87.0)		10 (43.5)		2.781 ± 0.195	
Body Mass Index based on Asia-Pacific classifications, n (%)						
Underweight	3 (50.0)	0.468	1 (16.7)	0.599	1.815 ± 0.396	0.852
Normal weight	18 (81.8)		9 (40.9)		2.393 ± 0.255	
Overweight	5 (83.3)		2 (33.3)		1.857 ± 0.428	
Obese	11 (78.6)		7 (50.0)		2.617 ± 0.347	
Comorbidities, n (%)						
Present	9 (56.3)	0.027	3 (18.8)	0.037	1.772 ± 0.371	0.017
Absent	28 (87.5)		16 (50.0)		2.702 ± 0.188	
Cardiovascular Comorbidities, n (%)						
Present	7 (53.8)	0.047	2 (15.4)	0.037	1.558 ± 0.316	0.038
Absent	30 (85.7)		17 (48.6)		2.685 ± 0.207	
Location, n (%)						
Colon	23 (71.9)	0.293	13 (40.6)	0.835	2.302 ± 0.245	0.237
Rectum	14 (87.5)		6 (37.5)		2.754 ± 0.249	
Side, n (%)						
Left	32 (78.0)	1.000	15 (36.6)	0.365	2.429 ± 0.209	0.523
Right	4 (80.0)		3 (60.0)		2.385 ± 0.381	
MSI, n (%)						
Microsatellite instability-High (MSI-H)/deficient mismatch repair (dMMR)	6 (54.5)	0.104	2 (18.2)	0.121	2.220 ± 0.209	0.427
Microsatellite stable (MSS) /proficient mismatch repair (pMMR)	16 (84.2)		10 (52.6)		2.466 ± 0.261	
Stage, n (%)						
III	9 (100.0)	0.169	6 (66.7)	0.137	2.679 ± 0.137	0.079
IV	27 (75.0)		13 (36.1)		2.303 ± 0.226	
Metastasis, n (%)						
Present	27 (75.0)	0.420	13 (36.1)	0.277	2.303 ± 0.226	0.072
Absent	9 (90.0)		6 (60.0)		2.679 ± 0.137	
Immune score, n (%)						
Low + Intermediate	20 (71.4)	0.319	10 (35.7)	0.517	2.215 ± 0.261	0.181
High	17 (85.0)		9 (45.0)		2.310 ± 0.193	

^a^ Calculated by Fisher’s Exact Test and Chi-Square Test for Independence.

^b^ Calculated by log-rank test (Mantel-Cox).

**Figure 1.**
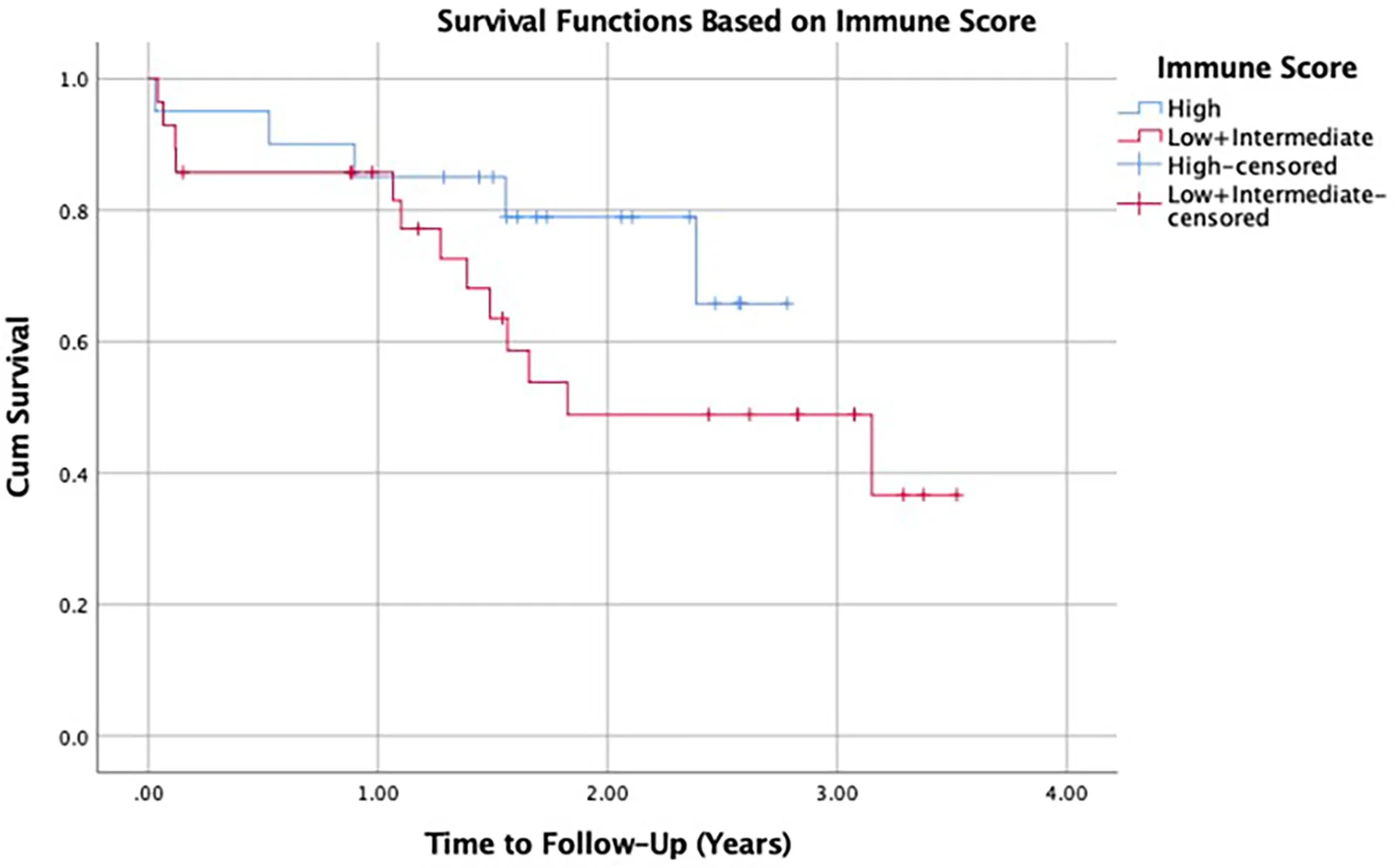
Kaplan-Meier curve based on immune score.

The survival curve indicated that CRC patients with elevated immune scores exhibited a less steep survival curve compared to those with low or intermediate immune scores ( Figure 1), indicating a higher survival rate at higher immune scores. However, there was no significant association between immune score and survival (p = 0.181, Table 3).

In the analysis of unadjusted and adjusted hazard ratios (HR) for mortality using Cox regression, several patient characteristics that were significantly associated with survival were examined ( Table 4). The analysis also included the immune score and variables previously shown to significantly influence the immune score. For patients with comorbidities, the unadjusted HR for mortality was 2.948 (95% CI: 1.165-7.462, p = 0.023), indicating a significantly higher risk compared to those without comorbidities. However, this association was not significant after adjusting for other variables. Similarly, patients with cardiovascular comorbidities had an unadjusted HR of 2.695 (95% CI: 1.018-7.132, p = 0.046), but this significance was not observed in the adjusted analysis. No significant associations were found for sex, immune score, stage, or metastasis in either unadjusted or adjusted HR analyses. These findings suggest that, while comorbidities and cardiovascular comorbidities initially appear to influence mortality, their effects are not significant when other factors are considered simultaneously.

**Table 4.** Unadjusted and adjusted hazard ratios for mortality of previously significant patient characteristics and immune score using Cox-regression analysis.

Patient characteristics	Unadjusted		Adjusted	
	HR (95% CI)	p-value	HR (95% CI)	p-value
Sex				
Male	2.812 (0.997-7.934)	0.051	2.567 (0.871-7.566)	0.087
Female	1		1	
Comorbidities				
Present	2.948 (1.165-7.462)	0.023	1.376 (0.270-7.010)	0.701
Absent	1		1	
Cardiovascular comorbidities				
Present	2.695 (1.018-7.132)	0.046	1.616 (0.293-8.911)	0.582
Absent	1		1	
Stage				
III	1	0.115	1	0.219
IV	5.108 (0.672-38.806)		3.742 (0.457-30.623)	
Metastasis				
Present	5.285 (0.696-40.114)	0.107	*	
Absent	1			
Immune score				
Low + Intermediate	2.012 (0.707-5.725)	0.190	1.299 (0.425-3.969)	0.646
High	1		1	

* Adjusted HR for metastasis was not obtained because of multicollinearity with the stage variable.

## 4. Discussion

### 4.1 Demographics of CRC patients

CRC is increasingly being diagnosed at earlier ages and more advanced stages, despite ongoing declines in overall incidence rates. ^9^ The majority of our patients diagnosed with CRC were below 70 years old (90.7%), with the highest at 40-69 years old. On the other hand, Rawla et al. stated that the majority of CRC are likely to be diagnosed over 65 years of age. ^10^ However, the number of cases has risen among people under 55 years old over the years, rising from 11% in 1995 to 20% in 2019. ^9^ This is a reversal of the trend of disease diagnosis at earlier stages, as seen from 1995 to 2005.

Despite the spread of early diagnosis in developed countries, the incidence of newly diagnosed cases were in advanced stages (60%) in 2019, whereas before the screening era, 52% of cases were diagnosed in advanced stages in the mid-2000s, and 57% in 1995. ^9^ This is in line with our study, in which all of our CRC patients were diagnosed at stage IV (78.4%).

The surge in CRC cases among younger individuals and the detection of more advanced stages reflects lifestyle and dietary shifts. Attributes such as increased ingestion of animal-based foods, sedentary habits, and obesity are independently linked to CRC risk. Lifestyle habits such as smoking, alcohol consumption, and intake of processed or red meat also contribute to this risk. ^9^ Conversely, diets rich in high-fiber foods, whole grains, dairy products, and calcium supplements are associated with a reduced risk of CRC. ^11^

Nearly half of our patients were classified as overweight (12.2%) or obese (32.4%) according to the Asian-Pacific BMI standards. Obesity is associated with a higher risk of CRC. ^12– 14^ Obesity is caused by a positive energy imbalance, which is characterized by an overall increase in caloric intake and a reduction in physical activity due to a sedentary lifestyle, in addition to hereditary predisposition.

A dose-response meta-analysis revealed that every 10 kg increase correlated with an 8% increase in CRC risk. ^15, 16^ Early life obesity predisposes individuals to a heightened risk of CRC in adulthood. ^12, 15^ This aligns with the existing literature highlighting elevated BMI (characterized by overweight and obesity) as a significant risk factor for CRC, although the precise molecular mechanisms remain elusive. Obesity triggers dysregulated lipid metabolism, altered adipokines and hormonal profiles, persistent inflammation, gut flora imbalances, and dysregulated bile acid equilibrium, all of which potentially fuel CRC carcinogenesis. ^17^ Notably, obesity triggers ongoing low-level inflammation through proinflammatory and anti-inflammatory cytokines originating from adipose tissue, such as IL-6, TNF-α, and PAI-1. ^18^ This chronic inflammation serves as a primary connection between obesity and the CRC tumor microenvironment, ^13^ activating pathways that drive proliferation, migration, and metastasis. ^19, 20^

Among our patients, 35.1% had comorbidities. The most common comorbidity was cardiovascular disease (28.4%), with hypertension being the most prevalent (20.3%). Other comorbidities included diabetes mellitus type 2 (8.1%), coronary arterial disease (5.4%), dyslipidemia (2.7%), hypertensive heart disease (1.4%), and arrhythmia (1.4%). Our results align with information obtained from the South Asia Cancer Registry, which showed that the most prevalent comorbidities of CRC are hyperlipidemia, hypertension, and diabetes. ^21^ CRC is typically prevalent in older individuals who often have chronic illnesses such as diabetes and cardiovascular diseases (CVDs). ^22^ Comorbidity rates are notably elevated among CRC survivors, ranging from 46% to 62%, ^23, 24^ surpassing those of control patients without cancer, which range from 11% to 50%. ^25^ Comorbidities exacerbate health challenges for cancer survivors, leading to increased symptom burden, reduced quality of life, elevated mortality rates, and heightened healthcare expenses compared with individuals without cancer. ^26^ Therefore, it is essential to implement specific actions and strategies for managing CRC because the prevalence of comorbidities is high among patients with CRC.

The Indonesian national guidelines recommend screening for CRC based on individual risk, personal preferences, and access. For those at moderate risk, screening should commence at age 50, incorporating rectal examination and a range of diagnostic modalities, including yearly fecal tests, such as fecal occult blood test (FOBT) or fecal immunochemical test (FIT). Colonoscopy, flexible sigmoidoscopy, CT colonography, and double-contrast barium enema should all be performed every five years. Patients with a history of polyps in previous colonoscopy, previous family history of CRC, and patients with a diagnosis of Hereditary Nonpolyposis Colon Cancer (HNPCC), Familial Adenomatous Polyposis (FAP), or Inflammatory Bowel Disease (IBD) were considered at high risk and should be recommended for colonoscopy based on the determined interval or time. ^26^ However, the CRC screening program in Indonesia has not been implemented across the entire population because of limited healthcare access and the lack of coverage for diagnostic procedures by the National Health Insurance for individuals with low to moderate risk. Primary prevention of CRC through screening remains key to reducing the burden of CRC. Screening of a large population cannot be achieved because of the significant cost of colonoscopy and other diagnostic procedures. Evidence suggests the use of guaiac fecal occult blood test (gFOBT) and fecal immunochemical tests as affordable and less invasive screening methods that can be implemented in Indonesia. ^1, 27^

We found that the majority of the patients were male (52.7%) slightly higher than females (47.3%). However, data from GLOBOCAN 2018 stated that the majority of CRC patients were male (1.5-fold higher than females). ^5^ In 2020, the global CRC incidence rate in males was 1,065,960, which is higher than that in females at 865,630. ^28^

A higher prevalence of left-sided tumors (86.5%) was observed compared to right-sided tumors (10.8%), ^29^ with tumor location playing a crucial role in disease progression, prognosis, and treatment strategies. Right-sided CRC, originating from the cecum, ascending colon, hepatic flexure, or transverse colon, exhibits distinct molecular characteristics and histology from left-sided CRC, which arises in the splenic flexure, descending colon, or sigmoid colon. ^29^ Disparities between the two sides of the colon are evident across physiological, molecular, and therapeutic domains. Notably, the National Comprehensive Cancer Network (NCCN) Clinical Practice Guidelines in Oncology underline variations in therapeutic responsiveness, particularly with EGFR inhibitors, such as cetuximab and panitumumab, which demonstrate greater efficacy as a first-line regimen for metastatic disease in left-sided CRC. ^30^ Additionally, patients with tumors on the right side often present with advanced tumor stages, larger tumor sizes, and more frequently poorly differentiated tumors, along with higher rates of lymphovascular invasion compared to those with left-sided CRC. ^31^ Systematic reviews and meta-analyses have associated right-sided CRC with poorer prognosis and decreased overall survival. ^32^

Tumors on the right side frequently possess mutations in the DNA mismatch correction mechanism, particularly in the microsatellite unstable group (MSI), and typically manifest flat histology. ^29, 33^ Despite this, the majority of patients with right-sided CRC in our study presented with adenocarcinoma histology (88.5%). Conversely, left-sided tumors are characterized by genetic alterations within the chromosomal volatility pathway, implicating genes, such as p53, BRAF, PIK3CA, APC, and KRAS. Patients with left-sided CRC derive greater benefit from adjuvant chemotherapies, such as 5-fluorouracil (5-FU)-based regimens, and targeted therapies, such as anti-epidermal growth factor receptor (EGFR) therapy, which contribute to a more favorable prognosis. In contrast, right-sided CRCs often exhibit poor responses to chemotherapy, but show more encouraging outcomes with immunotherapies, likely attributable to the heightened antigenic burden of these tumors. Recognizing right- and left-sided tumors as separate entities and tailoring treatment regimens is crucial for maximizing therapy effectiveness. ^29, 33^

Colonoscopy screening facilitates early detection of left-sided CRC, particularly in the form of small adenomas. Right-sided CRC can still be detected in the early stages; however, it is more challenging than left-sided CRC tumors because of their flat morphology. ^34, 35^ Therefore, right-sided tumors are more frequently identified in later stages than LCRC tumors. The majority of our patients had left-sided CRC (88.5%), however a large proportion of them were diagnosed with a late stage of disease, with 78.4% at stage IV and 14.9% at stage III. Notably, metastasis was present in the majority of patients, accounting for 78.4%). This is reflected in the low screening rate and poor early diagnosis of patients. This should be addressed to reduce the morbidity, mortality, and cost of CRC patients in our region.

The liver (52.7%), lung (21.6%), and peritoneum (10.8%) were the predominant sites of metastasis in CRC cases, consistent with findings from the Surveillance, Epidemiology, and End Results (SEER) database, which highlights the liver as the primary distant metastatic site, followed by the lung, bone, and brain. Given its adjacency to the colorectum, the liver is the most common anatomical site for CRC metastasis. ^36^

Determining the mutation status is crucial for prognosis and treatment options. Our data revealed that many patients did not have their mutation status evaluated, with only 70.3% checking for KRAS mutations, 66.2% for NRAS, 58.1% for BRAF, and 54% for MSI. The RAS and RAF protein families are key to signal transmission from growth factor receptors, promoting cell survival and proliferation. Overactivity of these pathways, mainly due to RAS and BRAF mutations, is common in various cancers. Women frequently develop right-sided colon cancer, which is typically linked to microsatellite instability and mutations in the BRAF gene. Conversely, men are more prone to left-sided colon cancer, which is often associated with chromosomal instability and KRAS gene mutations. Identifying KRAS, NRAS, and BRAF mutations is vital for determining patient eligibility for therapies such as panitumumab and cetuximab, as supported by clinical trials such as the PRIME study for panitumumab and the CRYSTAL study for cetuximab. ^37– 39^ Identifying mutations in KRAS, NRAS, and BRAF is crucial because the presence of these mutations results in a poor response to anti-EGFR therapy. Only 25.6% of patients with KRAS wildtype and left-sided tumors received cetuximab. The low utilization of cetuximab can be a result of several factors, including limited awareness, accessibility to targeted therapies, mutation testing, and its high cost. A significant number of patients (33.3%) had a high immune scores. However, no immunotherapy was administered to our patients because of the limited healthcare resources and high cost of the drug.

### 4.2 Relationship between patient characteristics and immune score

*4.2.1 Age and immune score*

The investigation into the association between age and immune score in patients with CRC revealed no statistically significant association (p = 0.768). Aging is known to induce changes in the immune system, especially in CD8+ and CD4+ T cells. There are molecular expression changes and functional alterations in T cells associated with aging, such as decreased responsiveness to new antigens, deficient migration patterns, and accumulation of memory and regulatory T cells. Moreover, alterations occur in the manifestation of co-stimulatory signals, including CD27 and CD28, which play a crucial role in activating T cells, along with a simultaneous increase in inhibitory molecules such as PD-1 and CTLA4, resulting in T cell exhaustion. ^40^ There are also sets of aging-related genes that are significantly activated in CRC tissues, predicting survival, severity, and the presence of immune cells within the tissue of CRC patients. ^41^ Older patients exhibit lower expression of CD3+ and CD8+ T cells at the invasive tumor edge and lower immune scores. ^42^ In breast cancer, the number of CD8+ cells weakly correlates inversely with patient age. ^43^

However, some studies have found that age is not a significant predictor of immune response in patients with CRC. Andric et al., who studied T cell subsets in patients with early onset CRC (aged <45 years) and average-onset CRC (aged 70-75 years) with left-sided colon and rectal locations, found no significant variances among the pair of groups concerning overall T cell infiltration, CD4+, CD8+, regulatory T cells, and γδ T cells. The expression of inflammatory mediators was also similar between the two groups. ^44^ Ugai et al. found no difference in immune cell density between patients with early- and late-onset CRC. ^45^ Lymph node ratio (LNR), a clinical hallmark denoting the immune reaction to colorectal tumors, is also strongly influenced by age. ^46^

Discrepancies in these study findings could stem from variations in sample size, ^44, 45^ patient demographics, CRC heterogeneity, including location, stage, and molecular subtype, as well as differences in the measurement of immune parameters between studies. The fact that there is an escalating incidence of CRC in adults under 50 is still unknown ^47^ and requires further research, including aspects of immune response parameters.

*4.2.2 Sex and immune score*

Sex was not associated with the immune score (p = 0.138). Barbosa et al. found no significant difference in the infiltration of CD3+ (p = 0.303), FoxP3+ (0.296), and CD8+ (p = 0.529) cells at the edges of CRC tumors between males and females. ^48^ Liu et al. reported similar findings for CD3+ (p = 0.254) and CD8+ (p = 0.714) cells. ^49^ The proximity scores of T cells between female and male patients with CRC did not differ significantly (p = 0.65). ^50^

However, some studies have reported conflicting results. Ray et al. found that females had higher T cell infiltration, specifically CD8, CD4, and Th2 cells, as well as IL-10+ macrophages, whereas males had higher levels of inflammation-related chemokines and cytokines. ^51^ Renman et al. concluded that higher total CD3+ and CD8+ scores were associated with the female sex. ^52^

The role of sex in the immune response in CRC patients is associated with several factors, including hormonal influences (lower levels of estrogen and progesterone in postmenopausal women connected with an increased number of CD4+ T cells within the tumor tissue) and differences in gene expression related to immune responses such as GARP, which modulates Treg cells in the colon, as well as CD96 and CCL14, which are associated with T cell activity and survival. Sexual dimorphism in the CRC microenvironment tends to favor females over males. ^53^

*4.2.3 Body mass index and immune score*

With a p-value of 0.905, there was no notable correlation between the body mass index (BMI) and immune score. When examining patients with CRC categorized by high and low BMI, no significant disparities were observed in gene regulation within CD8+ cells. ^54^ Hanyuda et al. discovered that lymphocytic response patterns (Crohn’s-like response, peritumoral response, intratumoral periglandular response, or tumor-infiltrating lymphocytes), overall lymphocytic ratings, and T cell density (CD3+, CD8+, CD45RO+, and FOXP3+) were not associated with BMI. Increased adiposity might be associated with an elevated risk of CRC, irrespective of the analyzed patterns of tumor lymphocytic infiltration. ^55^

Other studies have identified a connection between BMI and immune reactions in patients with CRC. Lavotshkin et al. determined that BMI exhibits an inverse relationship with CD3+ and CD8+ cells. ^56^ Ugai et al. concluded that long-term average BMI correlated with the emergence of tumors with low CD15+CD33- cell density, but not with tumors exhibiting medium or high CD15+CD33- cell density. ^57^

Obesity is known to increase the CRC risk by 2-3% for each incremental BMI unit. ^58^ Obesity is presumed to foster tumor development by disturbing antitumor immune reactions and provoking T cell fatigue via leptin-induced PD-1 elevation. ^59, 60^ The relationship between immune response and the correlation between obesity and CRC should be further investigated.

*4.2.4 Comorbidities and immune score*

There was no significant association between the existence or absence of comorbidities and the immune score (p = 0.362). Specifically, cardiovascular comorbidities were not associated with immune score (p = 0.267). Similar findings were obtained by Karjula et al. (p = 0.748) based on the Charlson Comorbidity Index (CCI). ^61^ The risk of CRC increases with the presence of cardiovascular risk factors, such as obesity (RR 1.31), active smoking (RR 1.20), diabetes (RR 1.25), and hypertension (RR 1.07). ^62^ The incidence of cardiovascular events, especially coronary heart disease, is higher in CRC patients within the first three years after diagnosis than in individuals without CRC. ^63^ However, research examining the influence of comorbidities, including cardiovascular comorbidities, on immune response parameters in colorectal tumors remains limited. ^64^

*4.2.5 Tumor location and immune score*

With a p-value of 0.947, there was no significant association between tumor location and immune score. Consistent with this, the baseline characteristics of the study by Koelzer et al. found no difference in the intraepithelial and stromal CD8 density between left-sided colon, right-sided colon, and rectal tumors. ^65^ Koelzer et al. found a non-significant link between CD8+ infiltrating the tumor and tumor location, namely, the colon and rectum (p = 0.355). ^66^ However, tumor location may be a consideration when assessing the prognosis of CRC based on immune response parameters, as different immune cells are specific prognostic factors for tumors at specific locations. An elevated presence of CD8+ cells serves as a positive prognostic predictor for patients with right-sided colon tumors situated on the right side. FoxP3+ concentration is an advantageous prognostic factor only for rectal tumors, whereas CD3+ is a favorable prognostic factor for right-sided colon and rectal tumors. ^67^

*4.2.6 Side and immune score*

The results of this study indicated no discrepancy in the immune score between left- and right-sided CRC (p = 0.439). CD8+ infiltration is more abundant in colon cancer tumors residing on the right side, ^49, 64^ while NK cell concentration is more profuse in left-sided CRC. Alongside the higher CD8+ infiltration on the right side, there is higher immune activation, as evidenced by the cytotoxic activity score, antigen presentation machinery, interferon-γ signature, and CD8+ T-cell/Treg ratio. The activation of NK cells in left-sided CRC is associated with longer survival rates. These differences in immune cell infiltration indicate why patients with tumors located on the right side exhibit a more favorable response to anti-VEGF antibodies, whereas patients with tumors situated on the left side show a superior response to anti-EGFR antibodies. ^64^ However, Guo et al. found conflicting results, where CD8+ expression at the invasive edge and immune score were inferior in right-sided tumors. ^68^ Berntsson et al. also found denser CD8+ infiltration in left-sided tumors. ^67^

Right-sided CRC is attributed to a 14% less favorable prognosis and diminished survival rates in contrast to cancer on the left CRC, with a 4.2% higher mortality rate. ^42, 69^ Left-sided cancer tends to be diagnosed earlier, possibly because it can be more easily diagnosed with sigmoidoscopy or with clearer symptoms, such as rectal bleeding or changes in bowel habits. ^69^ Perineural invasion and vascular emboli occur more frequently in right-sided cancers but are not associated with survival rates. ^42^ Further research is needed on the differences in the immune microenvironment between left- and right-sided cancers, considering that these differences may explain differences in prognosis and response to therapy agents between right- and left-sided cancers. ^64^

*4.2.6 MSI and immune score*

MSI was not associated with immune score (p = 0.238). This discovery diverges from previous research, which has indicated a link between MSI-high and immune score or heightened infiltration of cytotoxic lymphocytes, particularly CD8+. ^34, 70– 72^ Mlecnik et al. noted that the abundance of intratumoral CD8+ cells tends to be greater in MSI-high tumors, both within the tumor’s core and at its invasive periphery, whereas the CD8+ cell abundance in the stroma remains similar. ^73^

MSI-high tumors frequently exhibit numerous intraepithelial T cells in response to neoantigen presentation on the cell surface, potentially contributing to the enhanced prognosis of patients with MSI. ^74^ Immune score has been recognized as a more robust prognostic indicator than microsatellite instability status. ^73– 75^ Although the majority of patients with elevated immune scores also display MSI-high status, instances of high immune scores coupled with low MSI are not uncommon. In immunogenic MSI-low scenarios such as these, survival outcomes are more heavily influenced by the immune score rather than MSI status, as patients with elevated immune scores statistically exhibit prolonged survival compared to those with reduced immune scores, irrespective of their MSI status. ^74^

*4.2.7 KRAS, NRAS, and BRAF mutations*

There was no significant relationship between KRAS mutations and the immune score (p = 0.137). This aligns with Ogino et al., who identified no significant association between KRAS mutation status and T cell infiltration, although this study was limited to examining the density of specific T cell subsets. ^75^ Similarly, Kwak et al. reported that KRAS mutations are not linked to immune scoring models. ^76^ These findings suggest that while KRAS mutations contribute to an immunosuppressive TME, their impact on T cell infiltration may be nuanced and requires further investigation using comprehensive immune profiling techniques.

However, there are studies that have found a significant association between KRAS mutations and immune scores. KRAS mutations are prevalent in various cancers, including CRC, and are associated with distinct immunological signatures in the TME. Research indicates that colorectal cancers with KRAS mutations frequently demonstrate diminished infiltration of cytotoxic T cells and Th1 cells compared to their wild-type counterparts. ^34, 77, 78^ Additionally, CD4+ levels were lower in KRAS-mutant CRCs. ^79^ Furthermore, KRAS activation promotes an immunosuppressive TME by upregulating regulatory T cells (Tregs) and impairing the function of cytotoxic CD8+ T cells. ^80^ Mechanistically, mutant KRAS-expressing tumor cells release lactic acid, which sensitizes cytotoxic CD8+ T cells to apoptosis via NF-κB inhibition. ^77^ NF-κB and T-cell receptor signaling are inhibited in mutant KRAS CRCs cells. The immunosuppressive tumor microenvironment (TME) promotes the transformation of CD4+ cells into Tregs and upregulates the expression of CXCL3, which aids in the migration of Tregs into the tumor core. KRAS also decreases MHC-I molecules, causing CD8+ cells to be unable to recognize tumor cells. ^77, 80^ This immunosuppressive milieu mediated by KRAS mutations renders tumors less responsive to immune checkpoint inhibitors (ICIs) and adoptive T-cell therapies. ^77^

In this study, the association between immune score and mutations in NRAS and BRAF could not be determined because there were no cases of NRAS and BRAF mutations. Similar to KRAS mutations, NRAS mutations in CRC are associated with alterations in immune cell infiltration in the TME. CRCs harboring NRAS mutations exhibit significantly lower levels of CD4+ T cells than their wild-type equivalent. ^79^ However, the specific impact of NRAS mutations on immunotherapy efficacy remains unclear and warrants further investigation. In contrast to KRAS mutations, BRAF-mutant CRCs display increased immune cell infiltration, particularly higher levels of PD-L1 expression and CD8+ T cell infiltration, regardless of MSI status. ^81^ This immune-rich TME in BRAF-mutant tumors suggests a potential susceptibility to immunotherapy, including immune checkpoint blockade. Additionally, PD-L1 expression in CRC has been attributed to increased tumor-infiltrating lymphocytes and microsatellite instability, suggesting adaptive immune resistance. ^82^

*4.2.7 Stage and immune score*

Stage was significantly associated with the immune score (p = 0.040). Consistent findings were reported by Liu et al., who found significant correlations between CD3 and CD8 expression and the existence of metastasis in the lymph nodes as well as the III-IV TNM stage (p < 0.05). ^50^ Moreover, CRC at later stages, including those with lymph node infiltration, exhibited significantly diminished infiltration by CD8+ cells (p = 0.00004). ^83^ However, Irawan et al. revealed that the expression of CD8+ infiltrating the tumor did not differ significantly among stage I, II, III, and IV CRC (p = 0.390). ^66^ Similarly, Fachruddin and Jeo discovered no significant correlation between the immune score and clinical TNM stage of CRC (p = 0.640). ^84^

Studies have indicated that in early-stage CRC (stages I-II), CD8+ TILs are highly prognostic, suggesting a favorable outcome. ^85^ However, in metastatic CRC (stage IV), the impact of CD8+ TILs remains unclear. Saleh et al. examined the genetic activity of T cells across both stages of colorectal cancer, whether early or advanced. They found that CD8+ TILs from patients with advanced stages of CRC exhibited downregulation of genes linked to T-cell activation, migration, cell destruction, and adaptive immune response. ^86^ This downregulation suggests that CD8+ TILs in late-stage disease may have a diminished ability to migrate to tumor locations, dysregulated activation, and restricted responses to tumors. Furthermore, the upregulation of genes linked to cellular reactions to oxygen deficiency in advanced stages might result in programmed cell death of T cells and heightened release of inhibitory cytokines, such as IL-10. This dysfunction of cytotoxic T cells in advanced CRC stages may be associated with T cell exhaustion and differentiation into CD8+ regulatory T cells (Tregs). ^86^ In general, CD8+ TILs in later stages not only demonstrate dysregulated activation and effector functions but also exhibit a restricted capability to multiply and transform into memory T cells. Additionally, the prognostic significance of T-cell infiltration is affected by the expression of stromal cell-derived factor-1 (SDF-1). ^87^

*4.2.8 Metastasis and immune score*

There was a significant association between metastasis and immune score (p = 0.050). In CRC metastasis, immune score may vary. ^88^ However, Mlecnik et al. found that the immune score serves as a prognostic indicator of metastatic spread. ^89^ The presence of adaptive immunity and T cells has been linked to the prevention of premature metastatic infiltration. ^90^ In CRC metastases, the densities of CD3 and CD8 were lower (p < 0.05). In distant metastasis, the density of CD8 was lower (p = 0.025). ^91^ A lower density of CD8+ cells was positively correlated with lymph node metastasis (p = 0.042). ^92^ CD8+ T cells play a pivotal role in stopping tumor growth and hindering cancer metastasis. They achieved this by directly identifying and destroying cancer cells using specific intracellular antigens. In cancer, the expression of genes that inhibit CD8+ cells, macrophages, CD4+, and TH1 increases, thus forming an immunological environment that supports tumor defense and metastasis. ^93^

### 4.3 Relationship between patient characteristics and survival

The findings of this study highlight the significant influence of comorbidities, particularly cardiovascular, on patient survival. Patients with comorbidities had significantly lower one- and two-year survival rates and mean survival times than those without. These results align with those of previous studies by Michalopoulou et al. ^94^ and Pule et al., ^95^ which demonstrated that the presence of comorbidities negatively affected long-term survival and increased both CRC-specific and overall mortality. In particular, cardiovascular comorbidities significantly reduce survival rates. Janssen-Heijnen et al. reported increased mortality risk among older stage I or II colon cancer patients with cardiovascular disease. ^96^ Gouverneur et al. noted that while cardiovascular comorbidities impacted cardiovascular safety, they did not significantly affect the overall survival of patients treated with bevacizumab. ^97^ However, Cox regression analysis revealed that although comorbidities and cardiovascular comorbidities initially seemed to elevate the risk of mortality, their impact was not significant after accounting for other variables.

Regarding sex differences, the study found that females had a significantly longer mean survival time than males. This observation is supported by several studies, including those by Ma et al. ^98^ and Yang et al., ^99^ who found that females generally have better overall survival rates than males. However, it is important to note that other factors, such as tumor side and molecular characteristics, may also influence these outcomes, as highlighted by Baraibar et al. ^100^ However, Cox regression analysis did not show a significant association between sex and mortality in either unadjusted or adjusted HR analyses.

Survival analysis showed that CRC patients with moderate and elevated immune scores exhibited a less steep survival curve than those with low immune scores, suggesting better survival rates with higher immune scores. Nonetheless, the correlation between immune score and survival was not statistically significant (p = 0.181).

Research on CRC patients with liver metastases demonstrated that individuals with a high immune score experienced much longer recurrence-free survival (RFS) than those with a low immune score, with medians of 21.4 months versus 8.7 months, respectively (p < 0.001). The 3-year RFS rate was notably higher at 42.4% for high immune scores than at 17.0% for low immune scores (p < 0.001). For overall survival (OS), patients with high immune scores did not reach the median OS, whereas those with low immune scores had a median OS of 28.7 months (p < 0.001). Furthermore, the 5-year OS rate was a significantly different, with 59.7% for high immune scores versus 25.9% for low immune scores (p < 0.001). ^101^ Similarly, Li et al. revealed that high immune scores were significantly associated with better survival times (p = 0.048), with median survival at 101.4 months compared to 62.7 months for low immune scores. ^102^

This study did not find a relationship between immune score and survival, likely due to all patients were in advanced stages (III or IV), with 77% in stage IV, both of which are associated with poor survival outcomes. ^103, 104^ Although some studies have suggested that the immune score is prognostic in metastatic CRC (stage IV), Lin et al. found it ineffective in predicting outcomes for colorectal liver metastases after hepatectomy. ^105^ The immune score is currently validated primarily for stages I-III. ^5^

The relationship between mutation type and survival rate could not be assessed because no patients had NRAS or BRAF mutations. One patient with KRAS mutation had incomplete follow-up data. Studies have found that the overall survival of patients with KRAS mutations was lower than that of patients with wild-type KRAS (17 vs. 21 months, p = 0.002 in Kaplan-Meier survival analysis). ^106^ Rasmy, Fayed, Omar, and Fahmy also reported consistent result (19.6 vs 25 months, p < 0.001). ^107^

Elevated NRAS expression correlates with a risk that is 1.36 times higher for worse overall survival. ^108^ This aligns with the findings presented by Wang et al., where the risk was 1.83 times higher (p < 0.001). ^109^ Similarly, BRAF mutations are associated with a risk that is 1.52 times higher of diminished overall survival compared to BRAF with no mutations (18.9 versus 33.2 months, p < 0.001). ^110^ Notably, patients harboring wild-type KRAS, NRAS, and BRAF mutations exhibited median overall survival durations of 49.2, 36.2, 30.1, and 22.5 months, respectively (p < 0.001). ^109^

There are some limitations of this study. Several biases may potentially be present in this study and could, at least in part, influence the results. The relatively small sample size, including 74 patients in the current series, reduces statistical power, hence limiting the ability to detect significant associations-between variables like immune score and survival-that may be picked up when analyzing a larger cohort. In such a retrospective cohort, the selection bias may occur because available clinical data relies on clinical information that may not detect all relevant comorbidities of patients. This biases the data into the possible incompleteness of the follow-up data or underreporting of the comorbidities, hence giving biased results. The third is that the loss to follow-up was not accounted for, and the patients who were lost to follow-up were excluded while performing survival analysis, which again may create some bias by undervaluing mortality rates or overestimating survival in a cohort.

This study extensively investigated the demographic and clinical features of CRC patients, including age, sex, BMI, comorbidities, tumor characteristics, mutation status, immune score, disease stage, metastasis, and treatment. Among the 74 patients treated at Siloam Hospital, the majority were aged 41-70 years, male, and diagnosed with colon cancer. Comorbidities, mainly cardiovascular diseases, affected 36% of patients. Although cancer stage and metastasis were significantly associated with the immune score, no significant relationships were found between the immune score and survival. Although comorbidities were initially linked to survival rates, these associations lost significance after adjusting for confounding variables. Larger studies are warranted to confirm these findings and to elucidate the complex interplay between clinical parameters, immune responses, and survival outcomes in CRC.

#### Ethical considerations

This study was approved by the Ethics Committee of the Faculty of Medicine, Universitas Indonesia. The initial ethical approval number was KET-1096/UN2.F1/ETIK/PPM.00.02/2020, with approval date of September 28 ^th^, 2020,, which has been extended to 2025 with the approval number ND-166/UN2.F1/ETIK/PPM.00.02/2024, with approval date of February 28 ^th^, 2024. Considering the retrospective nature of this cohort study, the ethical approval committee waived the consent to participate as the research involved the analysis of existing data.

## Data Availability

OSF: Demographic profile of colorectal cancer patients in Indonesia: insights from a single-center experience and exploration of immune response and survival outcomes. https://doi.org/10.17605/OSF.IO/M9E85. ^111^ This project contains the following underlying data:
• Data file 1. MCRC Data_Cosphiadi Irawan_F1000 de-identified.xlsx Data are available under the terms of the Creative Commons Attribution 4.0 International Public License. This article follows *STROBE guidelines* for observational studies. OSF: Demographic profile of colorectal cancer patients in Indonesia: insights from a single-center experience and exploration of immune response and survival outcomes. https://doi.org/10.17605/OSF.IO/M9E85. ^111^ This project contains the following extended data:
• Data file 1. STROBE Checklist_Cosphiadi Irawan_CRC_F1000.docx • Data file 2. Cohort Flowchart_F1000_Cosphiadi Irawan.png Data are available under the terms of the Creative Commons Attribution 4.0 International license (CC-BY 4.0).
