# Supplementary material for: The Demographic Profile of Colorectal Cancer Patients in Indonesia: Insights from a Single Center Experience and Exploration of Immune Response and Survival Outcomes
Source: F1000Res. 2026 Apr 16;13:1362. Originally published 2024 Nov 14. [Version 2] doi: 10.12688/f1000research.155021.2 (PMC13550904; doi:10.12688/f1000research.155021.2)
Supplement: Peer review history

# Reviewer report: Reviewer response for version 2

**DOI:** 10.5256/f1000research.198163.r499220

**Published:** 2026-07-10

**Authors:** Leonardo Cárcamo

**Affiliations:** 1 Servicio de Cirugía, Hospital Base Valdivia, Valdivia, Chile

General Comments: This study tackles an important issue by evaluating the tumor immune microenvironment of colorectal cancer (CRC) in a resource-limited setting. However, there are several critical clinical and methodological red flags that make the current conclusions unreliable. The paper needs a major pivot before it can be considered for indexing.

**Major Critiques:**

- Biologically Improbable Mutation Rates: The reported KRAS mutation rate of 1.3% (and 0% for NRAS/BRAF) is the biggest issue here. Globally, KRAS mutations sit around 40-50%. A 1.3% rate in an advanced CRC cohort suggests a severe flaw in the PCR technique, data collection, or extreme selection bias. This anomaly invalidates the discussion on targeted therapies and must be addressed.
- Misapplied Immunoscore: The Immunoscore is a fantastic tool, but its validated prognostic value lies in early-stage disease (Stages I-III) to predict local recurrence. Because ~77% of this cohort is Stage IV, systemic disease burden completely eclipses the local immune response at the primary tumor margin. Finding no correlation with overall survival isn't a novel discovery; it's simply expected in this population.
- Statistical Overfitting: Running a multivariate Cox regression on an *N* of 74 with multiple covariates (plus up to 46% missing molecular data) guarantees statistical overfitting. The sample size simply doesn't have the power to support these complex survival models.
- Extreme Referral Bias: Reporting that 87% of the tumors are left-sided strongly implies a heavy referral or screening bias (e.g., lack of complete colonoscopies). Additionally, the survival analysis inappropriately lumps together highly variable treatments (curative resections vs. strict palliation) without adjusting for treatment intent.
- Discussion: The discussion reads too much like a literature review. Dedicating long paragraphs to generic topics like the mechanisms of obesity, NCCN guidelines, or the effects of NRAS/BRAF mutations dilutes the paper. The authors need to cut the fluff and focus strictly on interpreting their own data.

Conclusion: The current dataset doesn't have the statistical power nor the methodology to support complex survival or molecular hypotheses. The authors should rigorously audit their mutation data and reframe this manuscript as a purely descriptive, cross-sectional look at the reality of late-stage CRC presentations and immune profiles in a transitioning country.

Is the work clearly and accurately presented and does it cite the current literature?

Yes

If applicable, is the statistical analysis and its interpretation appropriate?

No

Are all the source data underlying the results available to ensure full reproducibility?

Partly

Is the study design appropriate and is the work technically sound?

No

Are the conclusions drawn adequately supported by the results?

Partly

Are sufficient details of methods and analysis provided to allow replication by others?

Partly

Reviewer Expertise:

colorectal cancer

I confirm that I have read this submission and believe that I have an appropriate level of expertise to confirm that it is of an acceptable scientific standard, however I have significant reservations, as outlined above.

# Reviewer report: Reviewer response for version 2

**DOI:** 10.5256/f1000research.198163.r476580

**Published:** 2026-06-01

**Authors:** Smreti Vasudevan

**Affiliations:** 1 Rajiv Gandhi Cancer Institute & Research Centre, Rohini, Delhi, India

The study is good and there are less studies on the topic from the authors’ part.

 After going through the manuscript, my comments are the following:

1. In the methods section the authors mention that follow-up period was from date of diagnosis/ treatment initiation. Were all the cases treatment naïve or treated cases also included. In the latter case can treatment cause the immune marker profiles to change?
2. Which software was used to measure CD3+ and CD8+ T cells?
3. Kindly provide raw calculation for sample size. If not in paper, then as comments for a better clarity. On what basis OS was presumed to be 75%?
4. There are some computing mistakes … for instance in Table 1: Male + Female sum comes 84 (not 74). Similarly, the table stats and written nos. show some differences. Please check the stats again and report identically.
5. Kindly mention the software used for statistical analysis, if used.
6. Is only stage III and IV cases included? If so then kindly include the same in inclusion criteria.
7. Generally, lost to follow up cases are censored. Why the authors have excluded those cases. Please explain.
8. Generally, right sided CRCs are considered more aggressive. The dataset includes more of left sided cases, more with low immune score. Was any survival benefit observed in the cohort due to it. Please explain in the context of existing literature.

Is the work clearly and accurately presented and does it cite the current literature?

Yes

If applicable, is the statistical analysis and its interpretation appropriate?

I cannot comment. A qualified statistician is required.

Are all the source data underlying the results available to ensure full reproducibility?

Partly

Is the study design appropriate and is the work technically sound?

Yes

Are the conclusions drawn adequately supported by the results?

Partly

Are sufficient details of methods and analysis provided to allow replication by others?

Partly

Reviewer Expertise:

Cancer Biology

I confirm that I have read this submission and believe that I have an appropriate level of expertise to confirm that it is of an acceptable scientific standard, however I have significant reservations, as outlined above.

# Reviewer report: Reviewer response for version 1

**DOI:** 10.5256/f1000research.170136.r356601

**Published:** 2025-01-20

**Authors:** Xishan Wang

**Affiliations:** 1 Department of Colorectal Surgery, National Cancer Center/National Clinical Research Center of Cancer/Cancer Hospital, Chinese Academy of Medical Sciences and Peking Union Medical College, Beijing, China

**General Overview:** The manuscript titled *"The Demographic Profile of Colorectal Cancer Patients in Indonesia: Insights from a Single Center Experience and Exploration of Immune Response and Survival Outcomes"* offers valuable insights into the clinical characteristics, immune responses, and survival outcomes of colorectal cancer (CRC) patients in Indonesia. The study provides an important contribution to the understanding of CRC in a specific geographic region, particularly in terms of tumor biology, immune responses, and comorbidities.

**Strengths:**

1. The study is comprehensive in covering key variables such as tumor characteristics, immune score, comorbidities, and genetic mutations.
2. The manuscript is well-organized, and the methods are clearly outlined, allowing for easy replication of the study.
3. The demographic analysis provides crucial data that can help guide clinical practice and future research, especially in low-resource settings.

**Areas for Improvement:**

1. **Study Design and Sample Size:**
  - The sample size of 74 patients may be too small to draw significant conclusions for some of the subgroups, especially given the retrospective design. Larger, multi-center studies would be beneficial to increase the generalizability of the findings.
  - Consider providing more justification for the study's sample size or discussing its limitations in greater detail.
2. **Statistical Analysis:**
  - The manuscript presents multiple p-values related to patient characteristics, immune scores, and survival, but the impact of confounders should be more clearly addressed. Specifically, comorbidities and their effects on survival could be explored in more depth.
  - The study's reliance on unadjusted hazard ratios for survival analysis might not fully account for confounding factors. A more robust multivariate analysis would strengthen the conclusions.
3. **Immune Score and Survival:**
  - Although the study finds a relationship between immune score and survival, the statistical significance is not strong (p = 0.181). It would be helpful to discuss potential reasons for the lack of significance and consider exploring other immunological markers or integrating more advanced immune profiling techniques.
  - Additionally, the absence of immunotherapy in the study population should be noted as a limitation in the interpretation of immune score-related survival data.
4. **Comorbidity Impact:**
  - The significant effect of cardiovascular comorbidities on survival is an important finding, but further discussion is needed on how these comorbidities interact with cancer treatment regimens and impact patient quality of life.
  - A deeper exploration of the mechanisms by which cardiovascular diseases influence CRC survival would add clarity to this section.
5. **Presentation of Data:**
  - Some sections of the results could be better summarized, especially in terms of the relationship between patient demographics, tumor characteristics, and immune scores. Tables and figures could be made more concise to enhance readability.
6. **Discussion and Conclusion:**
  - The discussion could benefit from a more thorough comparison with similar studies, particularly those conducted in other parts of Asia or low-resource settings, to highlight regional differences in CRC.
  - Recommendations for future research should be more explicitly stated, especially in regard to exploring the interplay between immune response, tumor mutations, and survival in CRC patients.

**Minor Points:**

- There are some typographical and grammatical errors in the manuscript. A thorough proofread is recommended before publication.
- Consider including a clearer figure legend for the Kaplan-Meier curve presented in Figure 1 to enhance clarity for readers.

**Conclusion:** Overall, the manuscript provides significant insights into the demographic and clinical characteristics of CRC patients in Indonesia. With some revisions, particularly in the areas of statistical analysis, sample size justification, and the discussion of immune scores, this manuscript has the potential to make a valuable contribution to the literature.

Is the work clearly and accurately presented and does it cite the current literature?

Yes

If applicable, is the statistical analysis and its interpretation appropriate?

Yes

Are all the source data underlying the results available to ensure full reproducibility?

Yes

Is the study design appropriate and is the work technically sound?

Yes

Are the conclusions drawn adequately supported by the results?

Yes

Are sufficient details of methods and analysis provided to allow replication by others?

Yes

Reviewer Expertise:

Colorectal cancer

I confirm that I have read this submission and believe that I have an appropriate level of expertise to confirm that it is of an acceptable scientific standard.

# Response

**Published:** 2025-01-23

**Authors:** Cosphiadi Irawan

**Affiliations:** Hematology and Oncology Medici, University of Indonesia, Depok, West Java, Indonesia

We appreciate the reviewer’s insightful comment regarding the sample size. We acknowledge that the retrospective design and the limited sample size of 74 patients may affect the statistical power of subgroup analyses, particularly for rarer mutation profiles, specific treatment modalities, or comorbidities. These analyses may require larger sample sizes to draw more robust conclusions.

 Our study focused on a single tertiary care center over a specific timeframe (2020-2022), which inherently limited the pool of eligible patients. The study included all eligible CRC patients treated during the study period, achieving near-complete case capture. The minimum sample size of 72 patients was determined based on an expected 3-year overall survival (OS) rate of 75%, with a 10% margin of error and a 95% confidence level, as described in the methods.

 We agree that larger, multi-center studies are essential to enhance the statistical power for subgroup analyses and to validate our findings across diverse populations. This limitation has been acknowledged in the discussion section of our manuscript.

 We also thank the reviewer for emphasizing the need to discuss the interaction between cardiovascular comorbidities, cancer treatments, and patient QoL. In response, we have expanded our discussion to address how cardiovascular conditions may limit treatment options, increase toxicity risks, and exacerbate side effects, collectively reducing QoL. We also highlight evidence linking cardiovascular comorbidities to functional limitations and higher symptom burdens.

 We appreciate the reviewer’s suggestion and have expanded the discussion to explore the mechanisms by which cardiovascular diseases influence CRC survival. Specifically, we now address how CVD limits treatment options, exacerbates systemic inflammation, and shares risk factors with CRC, which collectively contribute to poorer survival outcomes.

 Thank you for your valuable feedback. We have addressed the concerns as follows:

1. Confounders and Comorbidities: The influence of comorbidities and other confounders on survival has been discussed in the manuscript.
2. Multivariate Analysis: A multivariate analysis with adjusted hazard ratios for survival factors has been included in the study.
3. Immune Score and Survival: We have explained the lack of significance in immune score and suggested future research exploring additional immunological markers and advanced profiling techniques to better understand the immune landscape’s impact on survival.
4. Immunotherapy Limitation: The absence of immunotherapy in the study population has been noted as a limitation in interpreting immune score-related survival data.
5. Summary of Results: The relationship between patient demographics, tumor characteristics, and immune scores has been more clearly summarized.
6. Geographical Comparison: We have added studies to highlight geographical differences in CRC, particularly in low-resource settings.
7. Future Research Recommendations: A more explicit recommendation for future research exploring the interplay between immune response, tumor mutations, and survival has been added.
8. Proofreading: A thorough proofreading has been conducted to correct typographical and grammatical errors.
9. Figure Legend: The figure legend for the Kaplan-Meier curve in Figure 1 has been revised for clarity.
10. The response to your suggestions regarding the justification for the study's sample size limitation, how cardiovascular comorbidities interact with cancer treatment regimens and impact patient quality of life, and the exploration of the mechanisms by which cardiovascular diseases influence CRC survival has been provided in the previous response.

We hope these revisions address your concerns. Thank you again for your thoughtful suggestions.
